# Current Antibiotic Resistance Profile of ESKAPE Pathogens in a Nepalese Hospital: A Cross-Sectional Study

**DOI:** 10.1155/cjid/4426596

**Published:** 2025-03-27

**Authors:** Ranjit Kumar Sah, Abhinav Bhattarai, Priyatam Khadka, Sangita Sharma, Shyam Kumar Mishra, Junu Richhinbung Rai, Shristi Raut

**Affiliations:** ^1^Department of Laboratory Medicine, Institute of Medicine, Tribhuvan University, Kathmandu, Nepal; ^2^Biochemistry and Molecular Biology, SUNY Upstate Medical University, Syracuse, New York, USA; ^3^Department of Microbiology, Institute of Medicine, Tribhuvan University, Kathmandu, Nepal; ^4^Department of Microbiology, Tribhuvan University Teaching Hospital, Kathmandu, Nepal; ^5^School of Optometry and Vision Science, University of New South Wales, Kensington, New South Wales, Australia

## Abstract

**Background:** Antimicrobial-resistant *Enterococcus faecium*, *Staphylococcus aureus*, *Klebsiella pneumoniae*, *Acinetobacter baumannii*, *Pseudomonas aeruginosa*, *and Enterobacter* (ESKAPE) species pathogens pose a threat to global health by limiting available treatments, escalating the burden of disease, and raising mortality rates. This study investigated the prevalence of ESKAPE pathogens in different infections in a Nepalese hospital and studied their antibiotic resistance pattern.

**Methodology:** The study was performed from September 2022 to February 2023 at Tribhuvan University Teaching Hospital (TUTH), Kathmandu, Nepal. ESKAPE pathogens were isolated in accordance with standard procedures and subjected to antimicrobial susceptibility testing (AST). Identification was done via biochemical testing. The rates of multidrug resistance (MDR), production of extended-spectrum beta-lactamase (ESBL), and methicillin resistance were studied and statistically compared in terms of the type of pathogen, infection, and hospital admission.

**Result:** Altogether, 7429 different clinical samples were cultured and ESKAPE pathogens were isolated from 503/1564 (32.1%) positive samples. The prevalence of these pathogens was significantly higher in admitted patients (*p* < 0.001). Higher rates of isolation were from urine and sputum samples. *Klebsiella pneumoniae* was the most prevalent organism while *Enterobacter* was the least. A total of 52.3% and 7.4% of the isolates were MDR and ESBL producers, respectively. A significant proportion of MDR isolates were from patients admitted to the Intensive Care Unit (ICU). The prevalence of methicillin-resistant *Staphylococcus aureus* (MRSA) was 36.8%. AST revealed comparatively lower resistance of Gram-negative rods to tigecycline, polymyxin B, and colistin sulfate. Likewise, lower resistance rates to vancomycin and teicoplanin were observed in *S. aureus.*

**Conclusion:** In various clinical samples, we discovered that ESKAPE pathogens were more prevalent. In order to escape the ESKAPE's torment of antibiotic resistance, our findings urge the urgent implementation of sensible antibiotic use, training healthcare professionals in antibiotic stewardship, developing effective infection control strategies, and conducting effective surveillance.

## 1. Introduction

Despite significant advancements in medicine, combating antibiotic resistance has always been an evident challenge [1]. In less than 100 years of the discovery of the world's first antibiotic penicillin in 1928, today, healthcare has been running out of effective antibiotics due to the high emergence of resistant pathogens [2, 3]. Adapting and evolving against antibiotics, pathogens have incessantly emerged as multidrug-resistant (MDR) and hospital-acquired infections have been dominated by MDR strains [4]. In 2019, 5 million deaths occurred due to antibiotic resistant bacterial infection, and by 2050, the number is projected to reach 10 million, exceeding the current annual cancer deaths, thus potentially rising as the largest health problem in the time ahead [5, 6].

Amongst different pathogens evolving as resistant to antibiotics, *Enterococcus faecium*, *Staphylococcus aureus*, *Klebsiella pneumoniae*, *Acinetobacter baumannii*, *Pseudomonas aeruginosa*, *and Enterobacter* (ESKAPE) species group of pathogens have been marked as the ones with the highest mortality [7]. These pathogens are known to cause a fatal degree of bloodstream infections, pneumonia, meningitis, urinary tract infections, and pyogenic infections, leading to a significant proportion of mortality worldwide [8]. The World Health Organization (WHO) has listed these pathogens under the list of global priority pathogens that hold the urgency of novel and effective antibiotics so as to fight off their high MDR rates [9]. With consistency to the trend of outright antibiotic resistance, the Clinical and Laboratory Standards Institute (CLSI), therefore, has delisted from its list, the antibiotics that were previously tested for susceptibility [10].

ESKAPE pathogens have adapted and developed resistance to antibiotics via the development of different mechanistic approaches. Most common include the development of drug-inactivating mechanisms such as β-lactamases and aminoglycoside-modifying enzymes [11]. Likewise, modification of the target site, development of efflux pumps, and biofilm production have been effective armors against different antibiotics [12]. Importantly, the development of resistance by genetic mutations, mobile genetic element (MGE) acquisition, and their horizontal transfer among the circulating population has been inevitable [13]. Most strains circulating in the healthcare setting have developed the ability to produce biofilms, as a tool for adherence to indwelling medical equipment, therefore elevating the incidence of nosocomial infections [14]. It is indeed concerning from the findings of multiple studies on ESKAPE pathogens that they have not only represented a substantial proportion of the causation of all infections but have also been associated with increased length of hospital stay and a substantial spike in healthcare cost to the individual and nation as a whole [15].

Nepal is a developing country in South Asia with a high burden of infection from ESKAPE pathogens [16]. Poor infection control and irrational antibiotic prescription have been the intertwined reasons behind the emerging antibiotic resistance of these pathogens in the Nepalese context [17]. In this study, we intended to obtain the isolation rates of ESKAPE pathogens in different infections in a Nepalese healthcare setting, investigate its current antibiotic resistance trend, and discuss subsequent implications as well as mitigation measures.

## 2. Methods

### 2.1. Study Site and Ethical Approval

A cross-sectional study was performed at the Department of Microbiology, Tribhuvan University Teaching Hospital (TUTH), from September 2022 to February 2023. TUTH is one of the largest tertiary hospitals in Nepal with a high patient turnover and is known to dispatch efficient microbiological laboratory services. The ethical approval for this project was obtained from the Institutional Review Board of the Institute of Medicine, Kathmandu, Nepal (Approval number: 134 (6–11) E2 079/080).

### 2.2. Eligibility and Exclusion

All clinical specimens: urine, blood, sputum, swab, pus, body fluids, and tissues that were collected and transported in accordance with the guideline of the American Society for Microbiology (ASM) were included for the preliminary culture screening; only ESKAPE isolates were considered for AST. Organisms other than ESKAPE organisms were excluded. All specimens were processed within 2 h of collection. Specimens that exceeded 2 h of collection or those specimens unlabeled at the time of collection were excluded.

### 2.3. Specimen Processing and Culture

We followed the specimen processing protocol with regard to the type of specimen. Blood samples were collected on a 1:20 brain heart infusion broth and subcultured every 24 h. A negative culture was identified for no growth until 96 h of incubation at 37°C. Sputum samples were screened for adequate sampling based on Q-scoring and samples containing pus cells > 25/LPF, as well as epithelial cells < 10/LPF, were homogenized using dithiothreitol prior to culture. Urine, pus, and swabs were directly inoculated onto the culture medium. Body fluids were centrifuged and cultured.

### 2.4. Isolation and Identification

The eligible specimens were inoculated on nutrient agar, blood agar, MacConkey agar, and chocolate agar. Urine samples were inoculated on cysteine lactose electrolyte deficient (CLED) agar. Samples were incubated at 37°C for 24 h. After the incubation period, the isolated colonies were further subjected to identification. Reisolation was performed in cases of overlapping growth of different types of bacterial colonies. Identification was done preliminary by observation of colony morphology, hemolysis on blood agar, lactose fermentation on MacConkey and CLED agar, gram stain, oxidase, and catalase reaction. Identification was confirmed by means of coagulase test, and biochemical testing of citrate utilization, indole production, motility, urease production, along with specific characteristics on triple sugar iron (TSI) agar.

### 2.5. Antimicrobial Susceptibility Testing (AST)

AST was performed in accordance with Kirby–Bauer agar disk diffusion on Mueller–Hinton agar (MHA) (Oxoid, UK). Three to five pure isolated colonies of the same morphology were selected from the culture plate and transferred using an inoculating wire into a tube containing 5 mL of nutrient broth and then incubated overnight at 37°C. The broth was then diluted with sterile physiological saline to match with 0.5 opacity McFarland standard. A sterile swab stick was soaked into the broth, tipped off excess fluid, and lawn cultured on MHA plates. Commercially supplied antibiotic-impregnated filter paper discs (Biogram) were placed on the plates with 6 discs per plate at a distance of at least 24 mm center-to-center. After overnight incubation, the diameter of the zone of inhibition around each antibiotic disc was measured using a ruler and interpreted as sensitive, intermediate, or resistant as per the guideline of CLSI 2020 (M100).

### 2.6. Phenotypic Detection of ESBL Production and Methicillin Resistance

ESBL production and methicillin resistance were investigated in accordance with CLSI guidelines. The screening was done by observing the isolates' resistance to ceftriaxone (30 μg) disk. For isolates resistant to the antibiotic, confirmatory testing of ESBL production was performed by the measuring difference in the zone of inhibition diameters between ceftazidime (30 μg) and ceftazidime-clavulanate (30 μg–10 μg). Isolates with an increase of ≥ 5 mm in the zone of inhibition diameter around the clavulanate-containing disc were confirmed as ESBL producers. MRSA was confirmed if a zone of inhibition diameter ≤ 21 mm around cefoxitin (30 μg) disc was measured.

### 2.7. Data Management, Synthesis, and Statistical Analysis

The data on the types of specimen, patient demographics, and the number of isolates of each ESKAPE organism, antibiotic resistance, ESBL production, methicillin resistance, and MDR was tabulated on a prespecified data tabulation sheet on Microsoft Excel. The numbers were also expressed in terms of percentages. Statistical Analysis was performed on Statistical Package for Social Sciences (SPSS) Version 22, where the association of bacterial growth, the trend of antimicrobial susceptibility, ESBL production, and MDR rates between different patient categories and ESKAPE organisms isolated were compared using the Chi-square test.

A *p* value of < 0.05 was regarded as statistically significant.

## 3. Results

### 3.1. Demographics and Sample Distribution

Altogether, 7429 samples: 3846 urine, 1121 blood, 1255 sputum, 412 body fluids, 405 swabs, and 390 pus samples were received in the study duration. A total of 1564 samples were positive for significant growth among which ESKAPE pathogens were isolated from 503 (32.1%) clinical samples of different patients. The highest isolation rate of ESKAPE pathogens was observed in urine (45.5%), followed by pus (39.6%) and sputum (22.9%). There were 245 male and 258 female patients who were infected by ESKAPE pathogens and comprised 231 outpatients and 272 admitted patients. A majority of these patients ranged between 50 and 60 years old. Among the admitted patients, a substantial proportion (47.4%) from whom ESKAPE pathogens were isolated were admitted to the ICU. Altogether, the prevalence of individual organisms overall was as follows: *K. pneumoniae* (32.5%), *P. aeruginosa* (20.8%), *S. aureus* (18.8%), *E. faecium* (14.9%), *A. baumanii* (12.1%), and *Enterobacter* (0.9%).  Table 1 shows the demographic details of the study population, sample distribution, and organism distribution.

### 3.2. Distribution of ESKAPE Pathogens in Accordance With Specimens

**Figure 1:**
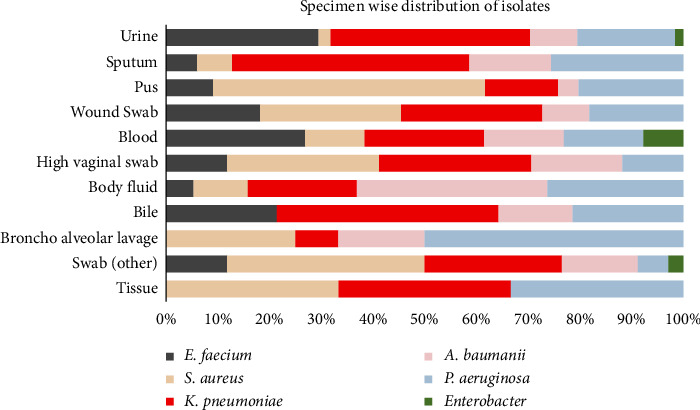
Specimen-wise distribution of isolated.

Figure 1 illustrates the distribution of different organisms isolated from various clinical specimens. *K. pneumoniae* was the predominant isolate in urine (38.6%), sputum (45.9%), and bile (42.9%). Pus and swab specimens were predominated by *S. aureus* (52.5% and 38.2%, respectively). There were equal rates of isolation of *K. pneumoniae* and *S. aureus* in wound swabs, high vaginal swabs, and tissue samples (27.3%, 29.4%, and 33.3%, respectively). Body fluid specimens were predominated by *A. baumanii* (36.8%). Half of the isolates from broncho alveolar lavage were *P. aeruginosa.* Blood culture showed the predominance of *E. faecium* (26.9%), followed by *K. pneumoniae* (23.1%).

### 3.3. Antibiogram of ESKAPE Pathogens

Table 2 displays the entire rates of susceptibility and resistance of individual organisms to respective antibiotics and has been elaborated in the following subheadings.

#### 3.3.1. *Enterococcus faecium*

Majority of *E. faecium* isolates were resistant to a number of drugs with high resistance rates to ciprofloxacin (97.3%), levofloxacin (90.7%), and doxycycline (93.3%). Similarly, a substantial proportion of isolates were resistant to high level gentamicin (70.7%) and erythromycin (83.3%). In contrast, there were no isolates resistant to vancomycin and teicoplanin. Highest rate of susceptibility following vancomycin and teicoplanin was observed for tigecycline (98.5%).

#### 3.3.2. *Staphylococcus aureus*

Highest proportion of *S. aureus* was resistant to penicillin (92.6%), followed by erythromycin (72.4%) and ciprofloxacin (61.1%). Similar to that of *E. faecium,* all *S. aureus* isolates were susceptible to vancomycin and teicoplanin. In addition, tigecycline and nitrofurantoin were susceptible in all isolates. A total of 83% of the isolates were susceptible to chloramphenicol. Unlike *E. faecium,* most *S. aureus* isolates were resistant to antibiotics in lower proportion.

#### 3.3.3. *Klebsiella pneumoniae*

There were no *K. pneumoniae* isolates resistant to polymyxin B and colistin sulfate. Resistance to tigecycline was 17.7%. Nearly half of the isolates were resistant to amikacin (48.1%). Apart from these antibiotics, all antibiotics were resistant in more than half a proportion of the isolates. Higher rates of resistance were observed for amoxicillin-clavulanic acid (95%), cefixime, and ceftazidime (81.5% in each).

#### 3.3.4. *Acinetobacter baumanii*

All isolates of *A. baumanii* were susceptible to polymyxin B and colistin sulfate. Majority of the isolates were resistant to a wide variety of antibiotics with higher resistance rates observed to amoxycillin-clavulanic acid (98.4%), chloramphenicol (95.7%), and aztreonam (93.4%), followed by cefixime (90.2%) and cefepime (80.3%). Bacteria showed a relatively higher susceptibility to tigecycline (55.6%); however, 44.4% were resistant to the antibiotic. Likewise, nearly half of the isolates were found to be susceptible to cefoperazone-sulbactam.

#### 3.3.5. *Pseudomonas aeruginosa*

We found that the susceptibility and resistance to antibiotics in the case of *P. aeruginosa* were equivalently distributed as nearly half of the isolates were resistant as well as susceptible to different antibiotics. A comparatively lower proportion of the isolates were resistant to cefoperazone-sulbactam (25.7%), piperacillin-tazobactam (30.5%), and cefepime (36.2%). There were no *P. aeruginosa* isolates resistant to polymyxin B and colistin sulfate.

#### 3.3.6. *Enterobacter* Species

All *Enterobacter* isolates were resistant to amoxycillin and cefixime. Likewise, higher rates of resistance were observed to ceftazidime, cefepime, and amoxycillin-clavulanic acid (80% each). In contrast, no resistance was observed to imipenem, meropenem, piperacillin-tazobactam, chloramphenicol, polymyxin B, and colistin sulfate.

### 3.4. Distribution of MDR, ESBL, and MRSA

**Figure 2:**
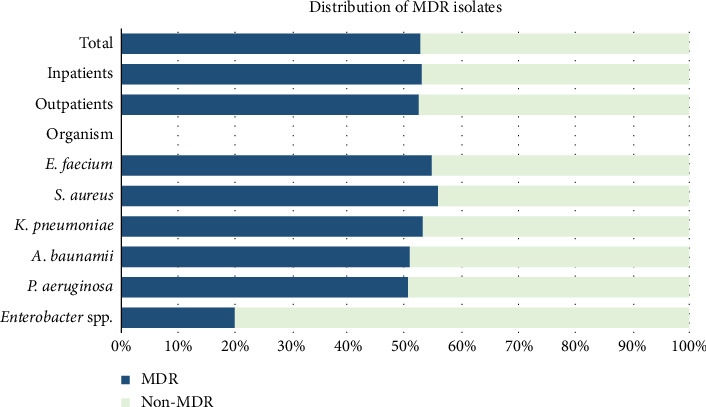
Distribution of MDR isolates.

Altogether, out of 503 isolates, 265 (52.7%) were MDR. More than half the proportion of the isolates in both inpatients and outpatients were MDR (52.4% and 52.9%). Among the inpatients, a substantial proportion of the MDR organisms were isolated from patients admitted to the ICU (47%). Except for *Enterobacter* whose rate of MDR was 20%, it was indeed concerning that the majority of the rest of the pathogens had significantly higher MDR rates (*p*=0.024). The proportions of MDR isolates were *E. faecium* (54.7%), *S. aureus* (55.8%), *K. pneumoniae* (53.1%), *A. baumanii* (50.8%), and *P. aeruginosa* (50.5%), which is illustrated in Figure 2.

Although the rate of MDR in *Enterobacter* was the lowest, the organism was found to possess the highest rate of ESBL production (20%). The rates of ESBL production were comparatively lower in other organisms: *K. pneumoniae* (8.6%) and *A. baumanii* (3.3%). No isolates of *P. aeruginosa* were ESBL producers. A total of 36.8% of all *S. aureus* isolates were MRSA and 18% showed inducible clindamycin resistance.

## 4. Discussion

Nepal, as a developing country, lacks sufficient sanitation and infection control standards that are required to tackle the high rate of infections [18]. In past years, with the aim of treating infections, the use of antibiotics in Nepal has sharply escalated which overflowed over the rational limits. Reports from Nepal's antimicrobial resistance surveillance have revealed that a majority of the patients had been unessentially prescribed more than one antibiotic [19]. Moreover, the practice of antibiotic prescription for febrile illnesses without any confirmation of underlying bacterial infection has been a major reason behind the emerging resistance [20]. The intent of self-medication among the general public and practices of dispensing antibiotics with no mandates on written prescriptions has further skyrocketed antibiotic abuse [21].

Our study investigated the prevalence of ESKAPE pathogens in a Nepalese hospital and explored its antibiotic resistance pattern. From our findings, the burden of ESKAPE pathogens in the Nepalese healthcare system has been evident. A total of 32.1% of all infections that we diagnosed were caused by ESKAPE pathogens and the highest isolation rates were observed in urine (45.5%), pus (39.6%), and sputum (22.9%). We observed a predominance of *Klebsiella pneumoniae* in urine (38.6%), sputum (42.9%), and bile (45.9%). Pus and swabs showed the highest isolation of *Staphylococcus aureus.* We observed equivalent isolation rates of *K. pneumoniae* and *S. aureus* from wound swabs, tissue samples, and high vaginal swabs. *Pseudomonas aeruginosa*, a popular hospital-acquired pathogen, was isolated in half of Broncho alveolar lavages. *Acinetobacter baumanii* predominated in body fluids. A substantial proportion of blood cultures revealed infection from *Enterococcus faecium.*

To closely evaluate the infection burden and emergence of antibiotic resistance in ESKAPE pathogens, we have compared our findings with that of Pandey et al. [16], a previous study addressing a similar issue in Nepal at a different hospital in 2018. The similarities of the prevalence and distinction of the antibiotic resistance rate of ESKAPE pathogens between theirs and our study at an interval of 5 years confer a concerning threat. Pandey et al. reported an 18.9% prevalence of ESKAPE pathogens out of all samples positive for growth, whereas the prevalence in our study was much higher (32.1%). Pandey et al. observed the highest isolation of ESKAPE pathogens from urine followed by pus and sputum and so did we. Similar to their findings, we found *K. pneumoniae*, *S. aureus*, and *P. aeruginosa* to be the commonest invaders; however, the study observed a predominance of *S. aureus*. The pattern of specimen-wise isolation of different organisms has been homogenous in both studies and the rise in prevalence as per our study advocates absolute evidence of the rising burden of ESKAPE organisms in infectious conditions.

The differences between the antibiotic resistance rates between the study in 2018 and our study have been evident. Amongst the Gram-positive cocci (GPC), we too observed the highest resistance to Ciprofloxacin, however, the resistance rate was higher in our study performed 5 years later the initial report (*E. faecium:* 92% vs. 97.3% and *S. aureus:* 58.3% vs. 61.1%). Vancomycin and teicoplanin were remarked as the most effective against GPC in both studies and the rational usage of these particular antibiotics should be highly prioritized to avoid resistance and secure uninterrupted efficacy. Likewise, in the case of Gram-negative bacilli (GNB), higher resistance rates were noted for cephalosporins, ciprofloxacin, cotrimoxazole, and amoxicillin-clavulanic acid. Surprisingly, the study of 2018 detected no *A. baumanii* urinary isolates resistant to nitrofurantoin, whereas our study as of 2023 observed a 73.3% resistance rate of *A. baumanii* to the antibiotic, therefore raising concern toward the emergence of nitrofurantoin resistance in the organism; a potential complication to the treatment of urinary tract infection from MDR strains of *A. baumanii*. Furthermore, the proportion of *Pseudomonas aeruginosa* resistant to different antibiotics has been noticeably high in our study. While less than half of the isolates in 2018 were resistant to ciprofloxacin, the numbers as per our findings have crossed to over half of the isolates. We found a rise in the resistance rates of *P. aeruginosa* to ceftazidime, cefepime, piperacillin-tazobactam, and amikacin (2018 vs. 2023: 29.8% vs. 47.6%, 28.6% vs. 36.2%, 8.3% vs. 30.5%, and 17.9% vs. 43.8%, respectively). Findings from both studies agree on polymyxin B and colistin being the most efficacious antibiotic for all GNB. Overall, we conclude from these observations that the antibiotic resistance of ESKAPE pathogens has indeed emerged in these 5 years and will potentially rise in years ahead unless irrational prescriptions are halted and novel antibiotics are developed.

Very importantly, we have noticed a significant difference in the MDR rate of ESKAPE pathogens. Pandey et al.'s study, which was performed in 2018, reported 43.3% of all ESKAPE isolates as MDR, whereas this rate in our study performed in 2023 was higher (52.7%). This finding is highly indicative of escalating antibiotic resistance of ESKAPE pathogens and the spike in the emergence of MDR strains in Nepal. When stratified to bacterial population, Pandey et al. reported the MDR rates of Gram-positive cocci (GPC) and Gram-negative bacilli (GNB) as 68% and 27%, respectively. In contrast, we found the MDR rates as 55.29% and 51.35% (GPC and GNB, respectively). We found a higher rate of isolation of MDR isolates among the admitted patients which could be explained by the high emergence and circulation of MDR strains within the healthcare setting [22]. In 2018, the rate of ESBL-producing *Enterobacter* was null, and currently, we identified 20% of the organism as ESBL producers. Paradoxical to the trend of evolving resistance from beta-lactamase, we found lower ESBL rates in ESKAPE pathogens as compared with Pandey et al., which might be indicative of the development of other resistance mechanisms equivalent to or outweighing the resistance due to hydrolyzing enzymes. The rate of methicillin resistance in our *Staphylococcus aureus* isolates was relatively lower than that in different studies [23–25].

Our study holds certain limitations. Susceptibilities for antibiotics including Vancomycin, Polymyxin B, and Colistin, were determined without the application of the minimum inhibitory concentration (MIC) technique. In addition, antibiotic resistance, ESBL, and MRSA were determined using phenotypic methods while the simultaneous molecular characterization of underlying resistant genes would add value for epidemiological surveillance. Due to low access to infrastructure, investigation on the rate of biofilm production and correlation with resistance profiles could not be performed. Since this was a single-center study the generalizability of our findings to the entire Nepalese population could not be fully achieved, therefore obscuring the pooled resistance pattern of the ESKAPE strains circulating in Nepal.

## 5. Conclusion

A substantial proportion of infections in our study accounted for ESKAPE pathogens. Specimens from hospital-admitted patients in our study harbored higher multidrug resistant strains, indicating a higher burden in the healthcare setting. Our findings advocate the urgency of implementing rational antibiotic use, educating healthcare professionals on antibiotic stewardship, formulating effective infection control strategies, and running efficient surveillance until novel interventions are available to escape the ESKAPE's torment of antibiotic resistance.

## Figures and Tables

**Table 1:** Patient demographics, sample distribution, and organism distribution of ESKAPE isolates.

	Inpatients	Outpatients	Total
*N* (%)	272	231	503

*Gender*			
Male	132	113	245 (48.7%)
Female	140	118	258 (51.3%)

*Age group*			
0–10	18	19	37 (7.4%)
10–20	12	19	31 (6.2%)
20–30	35	50	85 (16.9%)
30–40	30	30	60 (11.9%)
40–50	40	27	67 (13.3%)
50–60	62	27	89 (17.6%)
60–70	51	29	80 (15.9%)
70–80	20	22	42 (8.3%)
80–90	4	5	9 (1.8%)
90–100	0	3	3 (0.7%)

*Admitted units*			
ICU	129		
HDU	10		
Medical ward	48		272
Surgical ward	50		
Other	35		

*Specimens*			
Urine	48	84	132 (26.2%)
Sputum	109	24	133 (26.4%)
Pus	38	61	99 (19.7%)
Wound swab	8	3	11 (2.2%)
Blood	21	5	26 (5.1%)
High vaginal swab	3	14	17 (3.4%)
Body fluids	10	9	19 (3.7%)
Bile	8	6	14 (2.8%)
Broncho alveolar lavage	9	3	12 (2.4%)
Swab (others)	14	20	34 (6.8%)
Tissue	4	2	6 (1.3%)

*Organism*			
*Enterococcus faecium*	43	32	75 (14.9%)
*Staphylococcus aureus*	26	69	95 (18.8%)
*Klebsiella pneumoniae*	104	58	162 (32.5%)
*Acinetobacter baumanii*	34	27	61 (12.1%)
*Pseudomonas aeruginosa*	62	43	105 (20.8%)
*Enterobacter* spp.	3	2	5 (0.9%)

**Table 2:** Antibiogram of ESKAPE pathogens.

**Antibiotics**	***Enterococcus faecium* (*n* = 75)**		***Staphylococcus aureus* (*n* = 95)**	
	**Sensitive *n* (%)**	**Resistant *n* (%)**	**Sensitive *n* (%)**	**Resistant *n* (%)**

Amoxicillin (10 μg)	27 (36)	48 (64)		
Ciprofloxacin (5 µg)	2 (2.7)	73 (97.3)	37 (38.9)	58 (61.1)
Levofloxacin (5 µg)	7 (9.3)	68 (90.7)	67 (70.5)	28 (29.5)
Gentamicin (120 μg)	22 (29.3)	53 (70.7)		
Gentamicin (10 μg)			71 (74.7)	24 (25.3)
Nitrofurantoin (300 μg)	18 (43.9)	23 (56.1)	8 (100)	0 (0)
Erythromycin (15 μg)	4 (16.7)	20 (83.3)	24 (27.6)	63 (72.4)
Amoxicillin + clavulanic acid (10/10 μg)	29 (38.7)	46 (61.3)		
Piperacillin + tazobactam (100/10 μg)	31 (41.3)	44 (58.7)		
Tigecycline (15 μg)	66 (98.5)	1 (1.5)	95 (100)	0 (0)
Doxycycline (30 μg)	5 (6.7)	70 (93.3)	69 (72.6)	26 (27.4)
Vancomycin (30 μg)	75 (100)	0 (0)	95 (100)	0 (0)
Teicoplanin (30 μg)	75 (100)	0 (0)	95 (100)	0 (0)
Chloramphenicol (30 μg)	27 (75)	9 (25)	79 (83.2)	16 (16.8)
Cotrimoxazole (1.25/23.75 μg)			80 (84.2)	15 (15.8)
Penicillin (10 units)			7 (7.4)	88 (92.6)
Amikacin (30 μg)			78 (82.1)	17 (17.9)
Cephalexin (30 μg)			60 (63.2)	35 (36.8)
Cefoxitin (30 μg)			60 (63.2)	35 (36.8)
Clindamycin (2 µg)			49 (56.3)	38 (43.7)

**Antibiotics**	***Klebsiella pneumoniae* (*n* = 162)**		***Acinetobacter baumanii* (*n* = 61)**	
	**Sensitive *n* (%)**	**Resistant *n* (%)**	**Sensitive *n* (%)**	**Resistant *n* (%)**

Amoxicillin (10 μg)	0 (0)	162 (100)	1 (1.6)	60 (98.4)
Cotrimoxazole (25 μg)	46 (28.4)	116 (71.6)	19 (31.1)	42 (68.9)
Ciprofloxacin (5 µg)	43 (26.5)	119 (73.5)	16 (26.2)	45 (73.8)
Levofloxacin (5 µg)	66 (40.7)	96 (59.3)	17 (27.9)	44 (72.1)
Gentamicin (10 μg)	68 (42.0)	94 (58.0)	20 (32.8)	41 (67.2)
Amikacin (30 μg)	84 (51.9)	78 (48.1)	20 (32.8)	41 (67.2)
Cefixime (5 µg)	30 (18.5)	132 (81.5)	6 (9.8)	55 (90.2)
Ceftazidime (30 μg)	30 (18.5)	132 (81.5)	9 (14.8)	52 (85.2)
Nitrofurantoin (300 μg)	13 (23.2)	43 (76.8)	4 (26.7)	11 (73.3)
Amoxicillin + clavulanic acid (10/10 μg)	8 (4.9)	154 (95.1)	1 (1.6)	60 (98.4)
Imipenem (10 μg)	80 (49.4)	82 (50.6)	18 (29.5)	43 (70.5)
Meropenem (10 μg)	76 (46.9)	86 (53.1)	18 (29.5)	43 (70.5)
Cefoperazone + sulbactam (75/10 μg)	76 (46.9)	86 (53.1)	30 (49.2)	31 (50.8)
Piperacillin + tazobactam (100/10 μg)	75 (46.3)	87 (53.7)	21 (34.4)	40 (65.6)
Cefepime (30 μg)	40 (24.7)	122 (75.3)	12 (19.7)	49 (80.3)
Tigecycline (15 μg)	79 (82.3)	17 (17.7)	15 (55.6)	12 (44.4)
Polymyxin B (300 μg)	162 (100)	0 (0)	61 (100)	0 (0)
Colistin sulfate (10 μg)	162 (100)	0 (0)	61 (100)	0 (0)
Ampicillin + Sulbactam (10/10 μg)	70 (43.2)	92 (56.8)	29 (47.5)	32 (52.5)
Doxycycline (30 μg)	51 (31.5)	111 (68.5)	7 (11.5)	54 (88.5)
Chloramphenicol (30 μg)	46 (43.4)	60 (56.6)	2 (4.3)	44 (95.7)
Aztreonam (30 μg)	47 (29.0)	115 (71.0)	4 (6.6)	57 (93.4)

**Antibiotics**	***Pseudomonas aeruginosa* (*n* = 105)**		***Enterobacter* spp. (*n* = 5)**	
	**Sensitive *n* (%)**	**Resistant *n* (%)**	**Sensitive *n* (%)**	**Resistant *n* (%)**

Ciprofloxacin (5 µg)	47 (44.8)	58 (55.2)	2 (40.0)	3 (60.0)
Levofloxacin (5 µg)	52 (49.5)	53 (50.5)	3 (60.0)	2 (40.0)
Gentamicin (10 μg)	54 (51.4)	51 (48.6)	2 (40.0)	3 (60.0)
Amikacin (30 μg)	59 (56.2)	46 (43.8)	3 (60.0)	2 (40.0)
Ceftazidime (30 μg)	55 (52.4)	50 (47.6)	1 (20.0)	4 (80.0)
Imipenem (10 μg)	54 (51.4)	51 (48.6)	5 (100)	0 (0.0)
Meropenem (10 μg)	55 (52.4)	50 (47.6)	5 (100)	0 (0.0)
Cefoperazone + sulbactam (75/10 μg)	78 (74.3)	27 (25.7)	3 (60.0)	2 (40.0)
Piperacillin (10 μg)	51 (48.6)	54 (51.4)		
Piperacillin + tazobactam (10/10 μg)	73 (69.5)	32 (30.5)	5 (100)	0 (0.0)
Cefepime (30 μg)	67 (63.8)	38 (36.2)	1 (20.0)	4 (80.0)
Polymyxin B (300 μg)	105 (100)	0 (0)	5 (100)	0 (0)
Colistin sulfate (10 μg)	105 (100)	0 (0)	5 (100)	0 (0)
Amoxicillin (10 μg)			0 (0.0)	5 (100)
Cotrimoxazole (25 μg)			2 (40.0)	3 (60.0)
Cefixime (5 µg)			0 (0.0)	5 (100)
Nitrofurantoin (300 μg)			1 (50.0)	1 (50.0)
Amoxicillin + clavulanic acid (10/10 μg)			1 (20.0)	4 (80.0)
Ampicillin + sulbactam (10/10 μg)			4 (80.0)	1 (20.0)
Doxycycline (30 μg)			2 (40.0)	3 (60.0)
Chloramphenicol (30 μg)			3 (100)	0 (0.0)
Aztreonam (30 μg)			2 (40.0)	3 (60.0)

## Data Availability

The data used to support the findings of this study are available from the corresponding author upon reasonable request.

## References

[1] Chinemerem Nwobodo D., Ugwu M. C., Oliseloke Anie C. (2022). Antibiotic Resistance: The Challenges and Some Emerging Strategies for Tackling a Global Menace. *Journal of Clinical Laboratory Analysis*.

[2] Swann J. P. (1983). The Search for Synthetic Penicillin during World War II. *The British Journal for the History of Science*.

[3] Ventola C. L. (2015). The Antibiotic Resistance Crisis: Part 1: Causes and Threats. *P & T: A Peer-Reviewed Journal for Formulary Management*.

[4] Alemayehu T., Ali M., Mitiku E., Hailemariam M. (2019). The Burden of Antimicrobial Resistance at Tertiary Care Hospital, Southern Ethiopia: A Three Years’ Retrospective Study. *BMC Infectious Diseases*.

[5] Murray C. J., Ikuta K. S., Sharara F. (2022). Global Burden of Bacterial Antimicrobial Resistance in 2019: A Systematic Analysis. *The Lancet*.

[6] de Kraker M. E. A., Stewardson A. J., Harbarth S. (2016). Will 10 Million People Die a Year Due to Antimicrobial Resistance by 2050?. *PLoS Medicine*.

[7] Marturano J. E., Lowery T. J. (2019). ESKAPE Pathogens in Bloodstream Infections Are Associated With Higher Cost and Mortality But Can Be Predicted Using Diagnoses upon Admission. *Open Forum Infectious Diseases*.

[8] Denissen J., Reyneke B., Waso-Reyneke M. (2022). Prevalence of ESKAPE Pathogens in the Environment: Antibiotic Resistance Status, Community-Acquired Infection and Risk to Human Health. *International Journal of Hygiene and Environmental Health*.

[9] Asokan G. V., Ramadhan T., Ahmed E., Sanad H. (2019). WHO Global Priority Pathogens List: A Bibliometric Analysis of Medline-PubMed for Knowledge Mobilization to Infection Prevention and Control Practices in Bahrain. *Oman Medical Journal*.

[10] Mulani M. S., Kamble E. E., Kumkar S. N., Tawre M. S., Pardesi K. R. (2019). Emerging Strategies to Combat ESKAPE Pathogens in the Era of Antimicrobial Resistance: A Review. *Frontiers in Microbiology*.

[11] De Oliveira D. M. P., Forde B. M., Kidd T. J. (2020). Antimicrobial Resistance in ESKAPE Pathogens. *Clinical Microbiology Reviews*.

[12] Ramirez M. S., Tolmasky M. E. (2010). Aminoglycoside Modifying Enzymes. *Drug Resistance Updates*.

[13] Partridge S. R., Kwong S. M., Firth N., Jensen S. O. (2018). Mobile Genetic Elements Associated With Antimicrobial Resistance. *Clinical Microbiology Reviews*.

[14] Assefa M., Amare A. (2022). Biofilm-Associated Multi-Drug Resistance in Hospital-Acquired Infections: A Review. *Infection and Drug Resistance*.

[15] Tzouvelekis L. S., Markogiannakis A., Psichogiou M., Tassios P. T., Daikos G. L. (2012). Carbapenemases in *Klebsiella pneumoniae* and Other Enterobacteriaceae: an Evolving Crisis of Global Dimensions. *Clinical Microbiology Reviews*.

[16] Pandey R., Mishra S. K., Shrestha A. (2021). Characterisation of ESKAPE Pathogens With Special Reference to Multidrug Resistance and Biofilm Production in a Nepalese Hospital. *Infection and Drug Resistance*.

[17] Acharya K. P., Wilson R. T. (2019). Antimicrobial Resistance in Nepal. *Frontiers of Medicine*.

[18] Sarkar B., Mitchell E., Frisbie S., Grigg L., Adhikari S., Maskey Byanju R. (2022). Drinking Water Quality and Public Health in the Kathmandu Valley, Nepal: Coliform Bacteria, Chemical Contaminants, and Health Status of Consumers. *Journal of Environmental and Public Health*.

[19] Rijal K. R., Banjara M. R., Dhungel B. (2021). Use of Antimicrobials and Antimicrobial Resistance in Nepal: A Nationwide Survey. *Scientific Reports*.

[20] Landstedt K., Sharma A., Johansson F., Stålsby Lundborg C., Sharma M. (2017). Antibiotic Prescriptions for Inpatients Having Non-Bacterial Diagnosis at Medicine Departments of Two Private Sector Hospitals in Madhya Pradesh, India: A Cross-Sectional Study. *BMJ Open*.

[21] Mandal N. K., Rauniyar G. P., Rai D. S. (2020). Self-medication Practice of Antibiotics Among Medical and Dental Undergraduate Students in a Medical College in Eastern Nepal: A Descriptive Cross-Sectional Study. *JNMA; journal of the Nepal Medical Association*.

[22] Parajuli N. P., Acharya S. P., Mishra S. K., Parajuli K., Rijal B. P., Pokhrel B. M. (2017). High Burden of Antimicrobial Resistance Among Gram Negative Bacteria Causing Healthcare Associated Infections in a Critical Care Unit of Nepal. *Antimicrobial Resistance and Infection Control*.

[23] Shrestha A., Acharya J., Amatya J. (2020). Prevalence of ESBL and MBL Producing Gram Negative Uropathogens. *International Journal of Infectious Diseases*.

[24] Raut S., Bajracharya K., Adhikari J., Pant S. S., Adhikari B. (2017). Prevalence of Methicillin Resistant *Staphylococcus aureus* in Lumbini Medical College and Teaching Hospital, Palpa, Western Nepal. *BMC Research Notes*.

[25] Khanal A., Gaire A., Khanal A., Estrada R., Ghimire R. (2021). Methicillin-Resistant *Staphylococcus aureus* in Nepal: A Systematic Review and Meta-Analysis. *International Journal of Infectious Diseases*.

